# Predictive biomarkers and mechanisms underlying resistance to PD1/PD-L1 blockade cancer immunotherapy

**DOI:** 10.1186/s12943-020-1144-6

**Published:** 2020-01-30

**Authors:** Daixi Ren, Yuze Hua, Boyao Yu, Xin Ye, Ziheng He, Chunwei Li, Jie Wang, Yongzhen Mo, Xiaoxu Wei, Yunhua Chen, Yujuan Zhou, Qianjin Liao, Hui Wang, Bo Xiang, Ming Zhou, Xiaoling Li, Guiyuan Li, Yong Li, Zhaoyang Zeng, Wei Xiong

**Affiliations:** 1 NHC Key Laboratory of Carcinogenesis and Hunan Key Laboratory of Translational Radiation Oncology, Hunan Cancer Hospital and The Affiliated Cancer Hospital, Xiangya School of Medicine, Central South University, Changsha, Hunan China; 2 Key Laboratory of Carcinogenesis and Cancer Invasion of the Chinese Ministry of Education, Cancer Research Institute and School of Basic Medical Science, Central South University, Changsha, Hunan China; 3 Hunan Key Laboratory of Nonresolving Inflammation and Cancer, Disease Genome Research Center, The Third Xiangya Hospital, Central South University, Changsha, Hunan China; 4 Department of Medicine, Dan L Duncan Comprehensive Cancer Center, Baylor College of Medicine, Houston, TX USA

**Keywords:** Cancer immunotherapy, Immune checkpoint blockade, PD-1/PD-L1, Immune cells, Precision medicine

## Abstract

Immune checkpoint blockade targeting PD-1/PD-L1 has promising therapeutic efficacy in a variety of tumors, but resistance during treatment is a major issue. In this review, we describe the utility of PD-L1 expression levels, mutation burden, immune cell infiltration, and immune cell function for predicting the efficacy of PD-1/PD-L1 blockade therapy. Furthermore, we explore the mechanisms underlying immunotherapy resistance caused by PD-L1 expression on tumor cells, T cell dysfunction, and T cell exhaustion. Based on these mechanisms, we propose combination therapeutic strategies. We emphasize the importance of patient-specific treatment plans to reduce the economic burden and prolong the life of patients. The predictive indicators, resistance mechanisms, and combination therapies described in this review provide a basis for improved precision medicine.

## Background

Immunotherapy for cancer has unique advantages, including its precision and minimal side effects [1]. Tumor immunotherapy aims to eliminate tumors by enhancing the body’s own immunity. Tumors, on the other hand, evade attack by the immune system through a series of mechanisms known as “immune escape” [2]. The B7 family member, B7-H1 (PD-L1), plays an important role in this process [3–5]. PD-1, an immune checkpoint protein on T cells, binds to PD-L1 on tumor cells, promoting immune escape [6–8]. PD-1/PD-L1 blockade was a major breakthrough in cancer therapy. However, in many tumors, including non-small-cell lung cancer (NSCLC), renal cell cancer (RCC), and melanoma, PD-1/PD-L1 blockade therapy is only effective in a small proportion of patients [9]. Most patients do not respond to anti-PD-1 therapy (primary resistance), exhibit some initial sensitivity (adaptive resistance), or acquire resistance after relapse [10]; for example, one-quarter to one-third of patients with melanoma exhibit relapse and do not respond well to treatment (Table 1) [11]. Accordingly, resistance is a major limitation of anti-PD-1 therapy in clinical practice. To facilitate precision medicine and burden reduction in patients, we provide examples of curative effect biomarkers and resistance mechanisms against anti-PD-1 therapy. We further discuss combined treatments with the potential to improve efficacy.

**Table 1:** Representative FDA-approved immunological checkpoint inhibitors

Generic name	Trade name	Target	Application
pembrolizumab	Keytruda	PD-1	melanoma, non-small cell lung cancer, head and neck squamous cell cancer, classical Hodgkin lymphoma, primary mediastinal large B-cell lymphoma, microsatellite instability-high cancer, gastric cancer, cervical cancer, hepatocellular carcinoma, Merkel cell carcinoma, renal cell carcinoma, urothelial carcinoma
nivolumab	Opdivo	PD-1	metastatic small cell lung cancer, metastatic melanoma, metastatic urothelial carcinoma, metastatic colorectal cancer, hepatocellular carcinoma, metastatic nonsmall cell lung cancer, advanced renal cell carcinoma, classical Hodgkin lymphoma, metastatic squamous cell carcinoma of the head and neck
ipilimumab	Yervoy	CTLA-4	advanced renal cell carcinoma, adult and pediatric microsatellite instability-high or mismatch repair-deficient metastatic colorectal cancer, cutaneous melanoma, unresectable or metastatic melanoma
atezolizumab	Tecentriq	PD-L1	urothelial carcinoma, non-small cell lung cancer, triple-negative breast cancer, small cell lung cancer
avelumab	Bavencio	PD-L1	metastatic Merkel cell carcinoma, locally advanced or metastatic urothelial carcinoma [10]

### Predictive biomarkers of the efficacy of PD-1 blockade therapy

#### PD-1/PD-L1 expression

The combination of PD-1 and PD-L1 often leads to tumor immune escape [12]. Inhibiting immune suppression mediated by the PD-1 pathway is the basic principle of anti-PD-1/PD-L1 therapy. PD-L1 expression on tumor cells has high predictive value in melanoma and NSCLC and significance in angiosarcoma [13, 14]. In gastric cancer with high microsatellite instability (MSI-H), PD-L1 expression by immune cells is an important indicator of overall survival (OS) [15]. Decitabine improves the efficacy of anti-PD-1 therapy because PD-L1 in lung cancer cells is increased by IFN [16]. However, PD-L1 has the opposite effect when it exceeds a certain threshold; aromatic hydrocarbon receptor-induced PD-L1 overexpression in NSCLC reduces the efficacy of anti-PD-1 [17]. Valentinuzzi et al. found that patients with melanoma and moderate PD-L1 expression have the best response to anti-PD-1 therapy [18]. Furthermore, PD-1/PD-L1 levels may predict the efficacy of radiotherapy in head and neck cancers [19].

However, quantitative detection of PD-L1 as a prediction index requires antibodies and staining platforms [20–22], which contribute to differences in the accuracy of PD-L1 levels and may affect predictive value.

#### Antigen recognition initiates the immune response

The activation of adaptive immunity requires antigen recognition. Therefore, increased antigen recognition indicates a more active immune response [23]. The main predictors are MSI and tumor mutation burden (TMB).

Defective DNA mismatch repair (MMR) can cause MSI [24]. High MSI is associated with increased neoantigen production by tumors, greater immunogenicity, and stronger immune response. MSI is an excellent predictive biomarker, and the FDA has approved pembrolizumab to treat unresectable solid tumors with MSI-H or MMR defects (MMR-D) [25]. MSI frequency can also be used for tumor typing [26].

In a clinical trial of recurrent or metastatic colorectal cancer (CRC), patients with high MMR/MSI had better responses to immune checkpoint blockade [27]. MMR-D induction can reverse immunotherapy resistance in patients with pancreatic ductal adenocarcinoma [28]. The difference in MSI and the mutation load caused by MMR-D may explain differences in immunotherapy response. Efficacy is also related to the insertion-deletion mutation burden [29].

TMB, the total number of mutations per megabase in coding regions of tumor cells, is another predictor of therapeutic efficacy [30–32]. Patients with MSI-H tend to have a high TMB, and both parameters reflect instability in tumor cells. Whole exome sequencing can be used to measure exonic mutations in tumor cells comprehensively [33]. Keiichi et al. found that targeted genome sequencing can also be used to measure TMB [34]. TMB and other markers, including frameshifts and PD-L1 expression, are frequently used in clinical settings due to their strong correlation with anti-PD-L1/PD-1 drug effectiveness [35–39]. In intrahepatic cholangiocarcinoma with poor prognosis, patients with high TMB can even achieve complete remission with anti-PD-1 [40]. High TMB may indicate that new neoantigens can be produced by tumor cells to activate T cells suppressed by immune checkpoints [41, 42].

Similar to MMR proteins, POLE can repair errors caused by DNA replication. Mutant POLE is more easily detected by the immune system. Patients with endometrial carcinoma and POLE mutations have improved responses to treatment, and the POLE mutant subtype has better predictive value than the MSI subtype [43, 44]. However, effective methods to predict POLE mutations are needed.

#### Functional status of immune cells is related to anti-tumor immunity

Cytokines play important roles in the differentiation, maturation, and migration of various immune cells. Cytokine detection has predictive value for PD-1/PD-L1 therapy efficacy. Interferons and other cytokines are involved in killing or inhibiting tumor cells. TGF-β can inhibit the anti-tumor immune response and promote tumor cell escape. The blocking of TGF-β signaling can reverse insensitivity to anti-PD-1 therapy in CRC and prevent metastasis [45]. Similar results have been seen in bladder cancer [46]. Additionally, IFN-γ up-regulates major histocompatibility complex (MHC) II in antigen-presenting cells (APCs), enhances the production of CTLs, and up-regulates PD-L1 expression in tumor cells [47]. Its effects may be achieved via the JAK-STAT pathway [48]. IFN-γ is indispensable for anti-PD-1 treatment due to its role in the fragility of Tregs [49, 50]. Increased IFN can improve the efficacy of anti- pd-1 therapy [51]. High IFN-γ levels predict improved response to anti-PD-1 therapy in NSCLC [52]. Moreover, deficiency of IFN- signaling may cause tumor cells to resist other immune checkpoints [53]. Accordingly, IFN-γ levels may be used to screen patients who are likely to benefit from anti-PD-1 inhibitors.

Immunotherapy affects various cell and protein levels in the blood. These changes indicate immune cell status, which can predict the efficacy of immunotherapy. Significant changes in the percentage of KI-67^+^ cells among peripheral blood PD-1^+^CD8^+^ T cells predict long-lasting clinical benefits and prolonged progression-free survival in patients with thymic epithelial tumors [54]. Patients with melanoma and high C-reactive protein and absolute neutrophil counts (ANC) have a good response to treatment, and both parameters decrease after treatment [55]. However, unlike C-reactive protein levels, high ANC levels are not associated with better outcomes based on a large-scale analysis of clinical samples; when it exceeds a certain value (> 8000), prognosis is poor [56]. However, another study showed that reduced ANC after treatment is associated with cancer control [57]. The neutrophil-to-lymphocyte ratio (NLR) is often used to predict immunotherapy efficacy, and a lower baseline NLR is associated with better prognosis in patients with NSCLC and melanoma treated with nivolumab [56, 57]. Additional clinical trials are needed to identify predictive biomarkers in the blood.

### Infiltration of immune cells in the tumor microenvironment is a prerequisite for anti-tumor immunity

Activated T cell recruitment to tumor sites is necessary for their function in tumor cell killing. The efficacy of anti-PD-1 immunotherapy can be predicted according to the degree of immune cells infiltration, determined by two main factors: (1) chemokines (e.g., CCR5, CXCR3, CX3CR1, and CXCR6 are related to the migration of CTLs to tumor sites) and (2) entry through tumor blood vessels.

Tumor-infiltrating lymphocytes (TILs) differ from normal peripheral blood immune cells with respect to surface molecule expression, subtypes, and CD4^+^ and CD8^+^ T cell populations. PD-L1 expression differs significantly among tumors and is correlated with the distribution of invasive immune cells [58–61]. PD-L1 expression is positively correlated with TIL density in esophageal squamous cell carcinoma [62]. Anti-PD-1 therapy may be related to the degree of tumor-invasive immune cell infiltration, and an increase in local T cells can enhance anti-cancer effects [63]. High-density invasive CD8^+^ T cells are associated with prolonged OS in GC and CRC with ovarian metastases [64]. Induced T cell proliferation can relieve non-response to anti-PD-1 or PD-L1 therapy in pancreatic ductal adenocarcinoma [65]. In heterotypic tumor-stroma spheroids, the effect of blocking PD-1 can be increased by increasing TILs [66]. In limited clear cell RCC, two infiltrating T cell subtypes may be used to screen patients who may benefit from immunotherapy [67]. Recently, 37 genes in tumor-associated macrophages that differed between breast cancer tissues and healthy controls were candidate loci for predicting survival [68]. Interestingly, Jin et al. found that CD3^+^ T cells exhibit greater infiltration in PD-1^+^ tumors with MSI in Signet ring cell carcinoma, suggesting that there is a positive correlation between MSI and TILs [69]. Furthermore, EC with POLE mutations and MSI has more neoantigen and T cell infiltration, further demonstrating the association between these indicators and their value in predicting PD-1/PD-L1 blockade efficacy [44, 70].

**Fig. 1:**
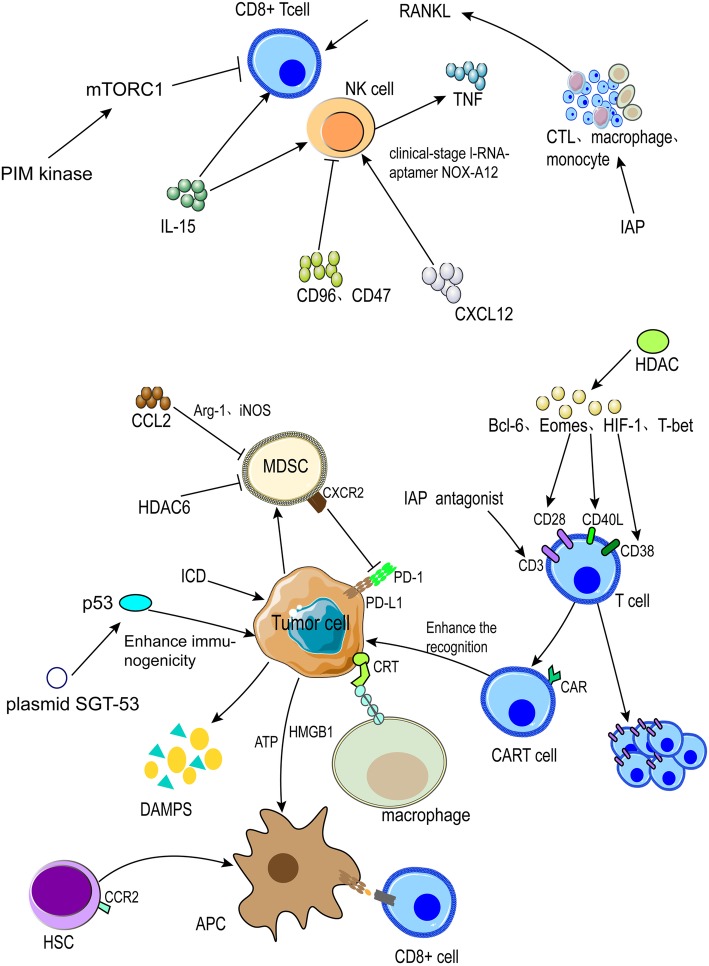
Summary of biomarkers of the response to anti-PD-1/PD-L1 immunotherapy. The efficacy of PD-1/PD-L1 blockade therapy is mainly predicted by PD-1/PD-L1 expression, microsatellite instability, tumor mutation load, and bone marrow-derived suppressor cells. The roles and significance of POLE, TGF-β, TGF-β, NLR, ANC, IDO1, and various chemokines are summarized. Biomarkers are shown in red

IDO1, another immune checkpoint protein, promotes the catabolism of tryptophan to inhibit T cells [71]. And IDO1 may be related to T cell infiltration [72]. Furthermore, anti-tumor T cells can be suppressed by Tregs and myeloid-derived suppressor cells (MDSCs) via IDO1, promoting tumor immune evasion [73]. In GIST and soft tissue sarcoma, activation of the IDO1 pathway causes immune suppression, decreasing the efficacy of anti-PD-1 therapy [74]. IDO1 has predictive value in some tumors and can be used to stratify and define some cancers [72, 75, 76]. These findings suggest that IDO1 is a good predictive biomarker and a new approach to cancer treatment (Fig. 1).

### Intestinal microbial flora affects host immune function

The intestinal microbiome plays a role in PD-1 blockade therapy. Bactericides can alter the effectiveness of anti-CTLA-4 treatment for melanoma [77]. Jin et al. found a strong correlation between the diversity of the intestinal microbiome and anti-PD-1 in advanced NSCLC. The gut microbiome may improve prognosis by increasing peripheral T and NK cells. Patients with melanoma and particular intestinal microbiome components may respond well to anti-PD-1 therapy. Increased efficacy of anti-PD-1 therapy has also been detected in sterile mice receiving fecal transplants from responsive patients [78, 79]. The intestinal microbiome may induce dendritic cell secretion of IL-12, increase CD4^+^ and CD8^+^ T cells, and promote TIL infiltration to improve the efficacy of anti-PD-1 in patients with melanoma [78, 80]. Progression-free survival and OS in the antibiotic treatment group were significantly shortened in advanced NSCLC, RCC, and urothelium carcinoma treated with PD-1/PD-L1 monoclonal antibody-based biotherapeutics [80]. The intestinal microbiome regulates the response to anti-PD-1 therapy, but the expression of PD-1 also affects the composition of the intestinal microbiome [81, 82]. Gastrointestinal immune-related adverse events, a common complication of anti-PD-1 therapy, disrupt the intestinal microbiome, which can lead to drug resistance [83, 84]. Routy et al. found a positive correlation between *Akkermansia muciniphila* and the efficacy of PD-1/PD-L1 blockade in lung cancer and RCC, and a positive response to immunotherapy in mice given oral bacterial supplementation [80]. Further research should focus on the detection of microbial taxa in the gastrointestinal tract with predictive value for anti-PD-1 responses and the use of fecal transplantation as an adjunct therapy.

### Mechanism underlying resistance to PD-1/PD-L1 blockade

#### T cell dysfunction-mediated resistance

Various processes, including recognition, activation, differentiation, and chemotaxis, are needed for T cells immune function. The disruption of one or several of these processes leads to T cell dysfunction and tumor immune escape. First, initial T cells must successfully identify tumor antigens presented by APCs. Next, the activation of primary T cells requires the antigen-MHC complex and the binding of B7 and CD28 on the cell surface, providing an important second signal. Finally, differentiated T cells migrate to specific tissues to perform immune functions and contribute to PD-1 blockade therapy resistance.

#### Antigen recognition disorders

**Fig. 2:**
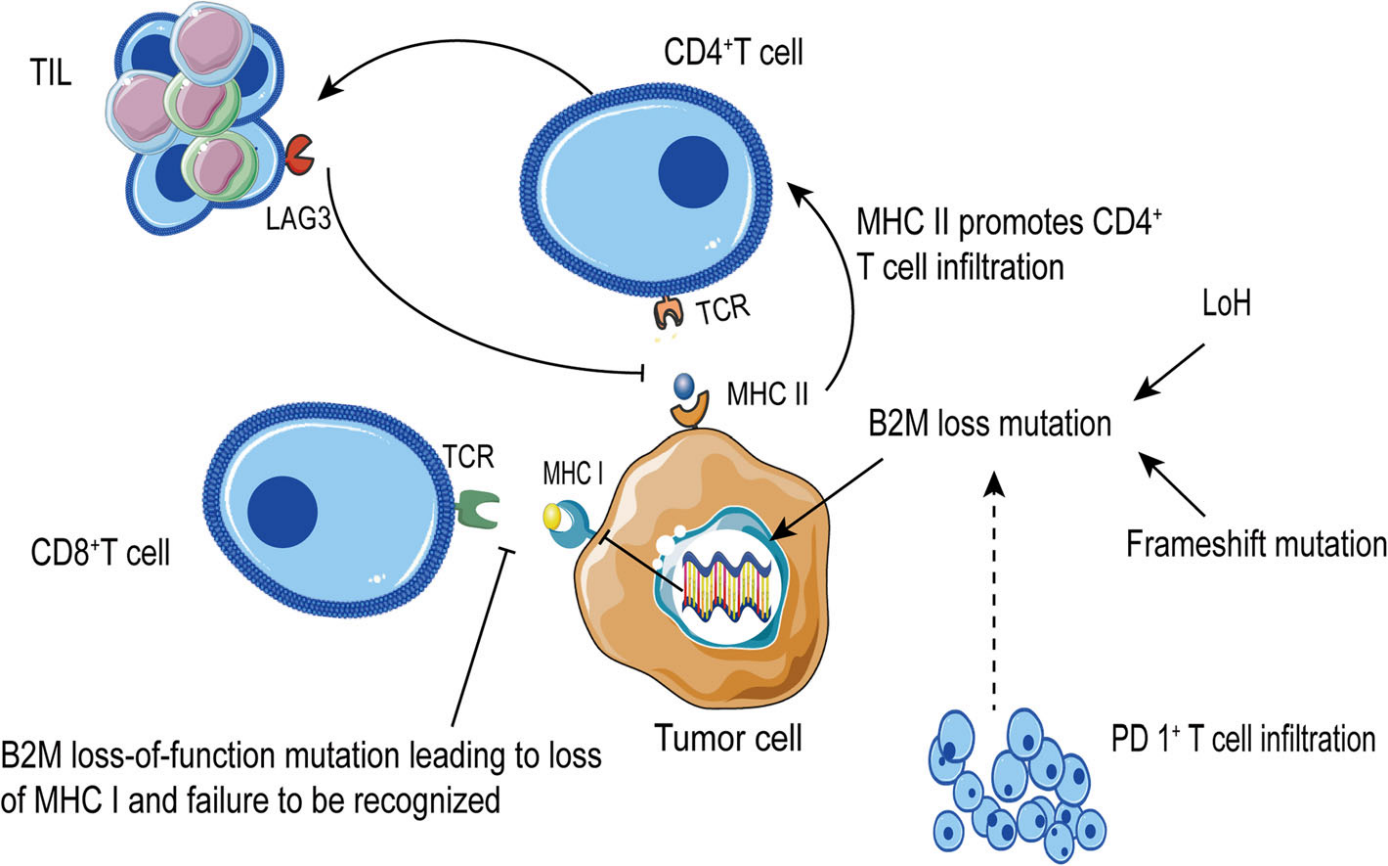
Anti-PD-1/PD-L1 immunotherapy resistance caused by antigen recognition disorders. Loss of heterozygosity and frameshift mutations in beta-2-microglobulin (B2M) disrupt tumor antigen presentation, and PD-1-positive T cell infiltration is associated with B2M. MHCII promotes CD4+ T cell infiltration and expresses the inhibitory receptor LAG3, which limits antigen presentation and causes primary resistance to PD-1 blockade therapy

Mutations in beta-2-microglobulin (B2M) disrupt antigen presentation, leading to immune checkpoint blockade therapy resistance. The deletion of B2M in animal models results in the deletion of HLA1 molecules, and approximately 29.4% of patients with progressive drug-resistant diseases have B2M abnormalities in clinical practice. Various mutations can result in a lack of tumor-specific B2M, especially a loss of heterozygosity. The B2M protein is an irreplaceable HLA1 molecule, and a lack of B2M negatively affects tumor antigen presentation and contributes to resistance to anti-PD-1 therapy [85–87]. Moreover, an increase in PD-1^+^ T cell infiltration is significantly correlated with an increase in B2M mutations, indicating that drug resistance caused by B2M mutation is associated with PD-1^+^ T cell infiltration [88]. In addition to B2M mutations, limited antigen presentation is related to the autonomous expression of MHCII. In MHCII^+^ tumor microenvironments, the infiltration of CD4^+^ T cells increases and LAG3 (an MHCII inhibitory receptor)-induced TIL expression increases, thereby limiting antigen presentation and promoting resistance to anti-PD-1 therapy (Fig. 2) [89, 90].

#### T cell activation disorders

Shayan et al. found that after blocking PD-1/PD-L1, TIM-3 expression, another immune checkpoint, is upregulated, inhibiting the activation of T cells by inhibiting the phosphorylation of AKT/S6, leading to a decreased immunotherapeutic response [91]. TNF is essential for the expression of TIM-3 in TILs, and its compensatory expression is upregulated after blocking PD-1, thereby inducing TIM-3 expression [92]. In melanoma, anti-PD-1 treatment also increases the inhibitory immune checkpoint, VISTA, that synergistically inhibits T cell activation with PD-L1, leading to adaptive resistance; its expression is higher than that of PD-L1 in CRC [93].

Furthermore, changes in specific genes can also cause T cell activation disorders. Up to one-third of melanomas are accompanied by PTEN deletion, for which the mechanisms include gene mutations and deletions, loss of chromatin, loss of heterozygosity, and epigenetic changes such as hypermethylation-induced transcriptional silencing [94–100]. PTEN itself negatively regulates the PI3K/AKT pathway and down-regulates PD-L1 expression. In melanoma, PTEN deletion promotes AKT phosphorylation, thereby promoting PI3K/AKT pathway activation, and ultimately promotes PD-L1 expression, thereby inactivating T cells. Additionally, PTEN inhibits the expression of immunosuppressive factors IL-10, IL-16, and VEGF through the PI3K/AKT-dependent pathway, and its deletion promotes the activation of the PI3K/AKT pathway, thereby activating STAT3 and eventually increasing IL-10, IL -16, VEGF, and CCL2. Meanwhile, PTEN inhibits the production of the proinflammatory cytokine IL-12 by dendritic cells, forming a suppressive immune microenvironment that inhibits the activation of T cells [94, 101]. In glial tumors and glioblastomas, PTEN deletion activates the PI3K/AKT-mTOR pathway by promoting the activation of ribosomal protein S6 kinase β-1 (S6K1), thereby promoting PD-L1 translation. Thus, PTEN deletion also deactivates T cells [102].

**Fig. 3:**
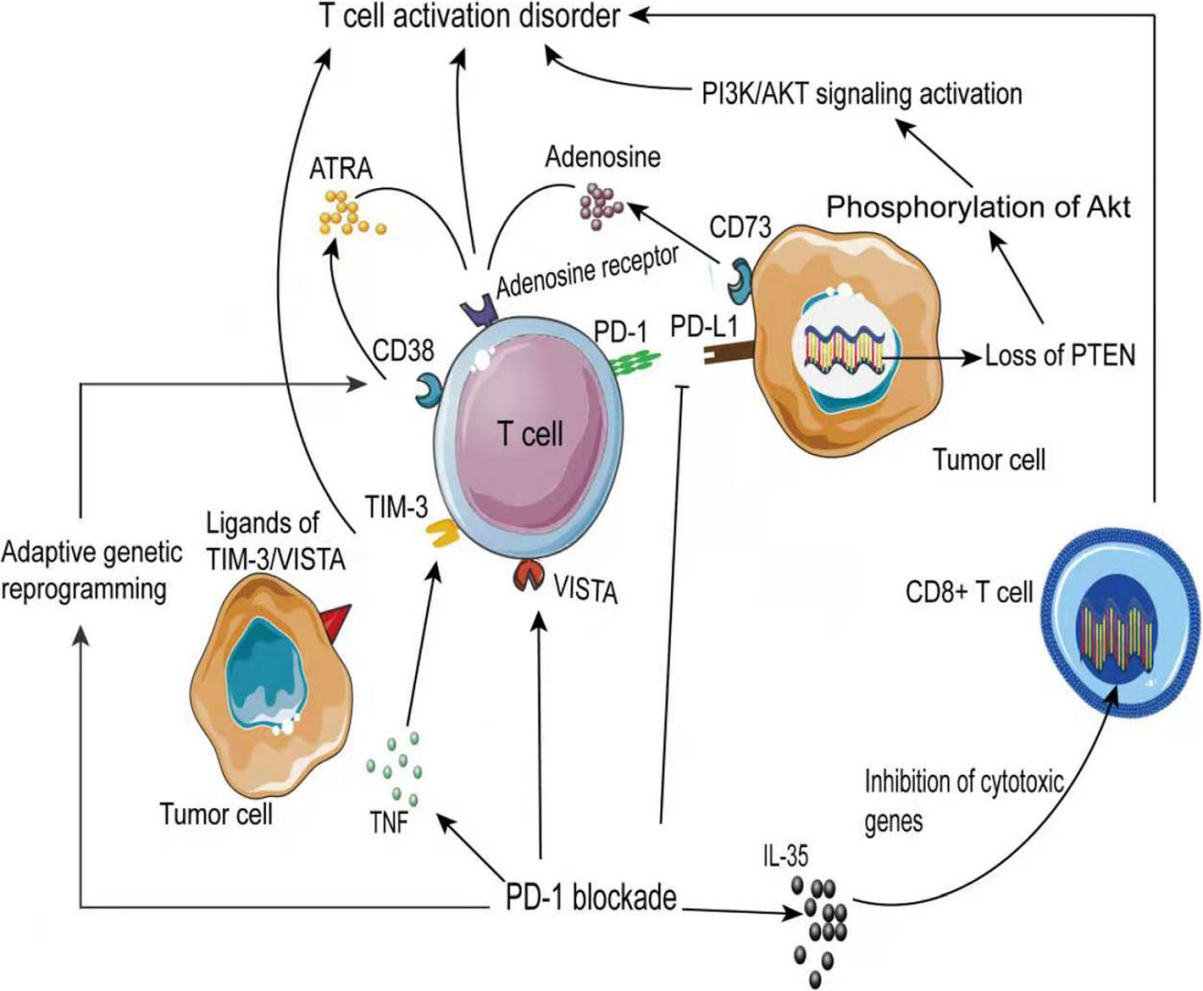
Inhibiting T cell activity causes anti-PD-1/PD-L1 immunotherapy resistance. After PD-1 blockade, the secretion of cytokines, including TNF and IF-36, causes T cell gene alterations, which inhibits cytotoxicity, promotes TIM-3 and VISTA inhibitory checkpoint expression, up-regulates CD38, and promotes ATRA secretion and binding to adenosine receptor and adenosine inhibition of T cell activation. The deletion of PTEN in tumors activates the PI3K/AKT pathway through multiple routes, including phosphorylation of Akt and activation of S6K1, to promote PD-L1 expression and inhibit T cell activation

When PTEN is silenced, PI3K pathway blockade can reduce the activation of AKT, thereby relieving resistance to anti-PD-1 therapy [94]. The blockade of PD-1/PD-L1 results in the adaptive reprogramming of genes in the tumor immune microenvironment, where the up-regulation of CD38 on T cell surfaces leads to resistance [103]. CD38 activation of adenosine receptors by all-trans-retinoic acid (ATRA) inhibits T cell function via adenosine expression [103]. Because adenosine is a strong immunosuppressive substance, it inhibits effector T cell immune function by cytokine secretion and inhibits T cell proliferation [104]. CD73 binding to adenosine receptor 2A on T cells produces adenosine, inhibiting the immune response to PD-1/PD-L1 blockade [105]. Some interleukins have a negative regulatory role in T cell function. IL-35 inhibits the expression of cytotoxic genes in CD8^+^ T cells and reduces cytolytic and noncytolytic functions [106]. Recent studies have shown that the Notch signaling pathway may inhibit FASL and perforin, resulting in decreased activity and dysfunction of CD8^+^ T cells (Fig. 3) [107].

#### Decrease in T cell infiltration

**Fig. 4:**
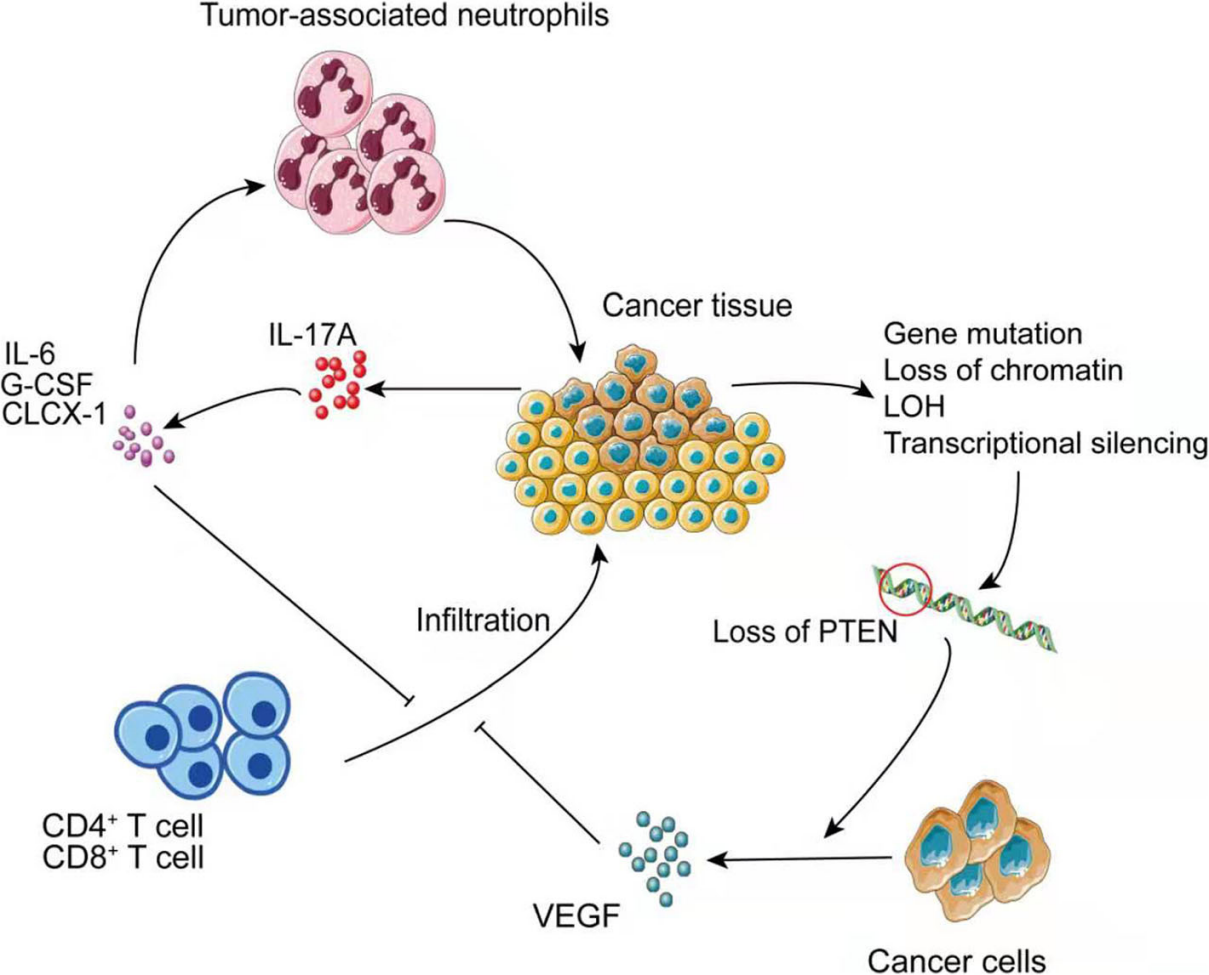
Reduced T cell infiltration leads to drug resistance. The secretion of IL-6, G-CSF, and CXCL1 promotes the migration of tumor-associated neutrophils to tumor tissues and inhibits the entry of CD4+ and CD8+ T cells into the tumor microenvironment, whereas PTEN deletion caused by different mechanism up-regulates VEGF expression, which promotes tumor angiogenesis, leading to impaired perfusion and decreased CD8+ T cell infiltration. Moreover, in some solid tumors, it is difficult for T cells to pass through an immunosuppressive tumor stroma, resulting in resistance to PD-1 blockade

A decrease in effector T cells in the tumor microenvironment also contributes to resistance to anti-PD-1 therapy. Tumors are characterized by the upregulation of IL-6, granulocyte colony-stimulating factor (G-CSF), and CLCX1 by increasing IL-17A expression. IL-6 promotes tumor proliferation. G-CSF increases tumor-associated neutrophils and decreases CD4^+^ and CD8^+^ T cells in the tumor microenvironment. IL-17A^+^ tumor tissues are also significantly less reactive to PD-1 antibodies in clinical samples [108]. Additionally, the absence of PTEN increases VEGF expression. Elevated VEGF promotes abnormal tumor angiogenesis, which reduces perfusion in blood vessels, causing a hypoxic environment and inhibiting T cell infiltration [109–112]. Therefore, the absence of PTEN may reduce the infiltration of CD8^+^ T cells by upregulating VEGF, leading to resistance to PD-1 therapy [94]. MDSCs are negatively correlated with CD4^+^ and CD8^+^ T cell infiltration and are an important factor in decreased T cell infiltration [113]. Additionally, the presence of immunosuppressive tumor stroma, especially in some solid tumors, makes it difficult for T cells to infiltrate, limiting the efficacy of PD-1 blockade immunotherapy. Irreversible electroporation of the tumor matrix can address this issue [114]. Therefore, immunosuppressive tumor stroma should be studied further (Fig. 4).

#### T cell depletion leads to resistance to PD-1 blockade therapy

**Fig. 5:**
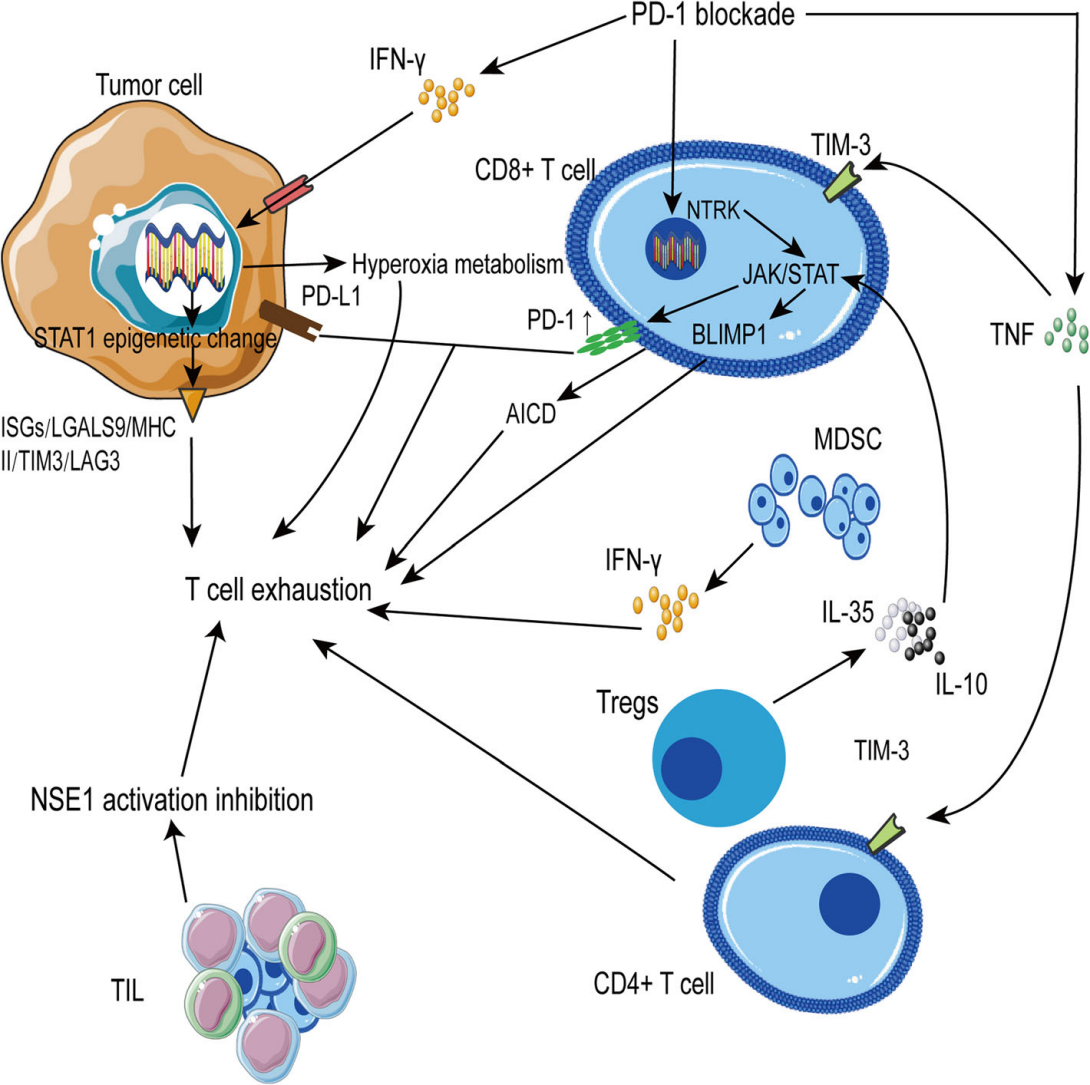
T cell exhaustion causes PD-1 blockade therapy resistance. PD-1 blockade promotes the secretion of cytokines, including IFN-γ and TNF, leading to the expression of ligands of inhibitory receptors, including LAG3 and TIM-3, in tumor cells and activation-induced cell death (AICD). Additionally, PD-1 blockade can attenuate the expression or activity of a series of genes and promote T cell exhaustion. Furthermore, after PD-1 blockade, tumor cells show high oxygen consumption, which causes hypoxia in the tumor microenvironment, promoting the exhaustion of T cells. Moreover, NSE1 activity in TILs is inhibited, which affects glycolysis and leads to T cell depletion

T cells play a major role in tumor immunity but, in long-term diseases, the dysregulation of T cell subsets or decreases in mature T cells can occur, known as “T cell depletion” [115]. Many mechanisms explain this process, including increased co-inhibitory receptors on T cell surfaces and epigenetic changes in memory T cells. In anti-tumor immunity, chronic persistent type II interferon signaling enables STAT1 tumor-related epigenetic changes, resulting in increased expression of interferon-stimulated genes and inhibitory receptors (TCIRs) on multiple T cells, including *LGALS9* (Galectin-9), MHCII ligands, and immune inhibitory checkpoints, including TIM3 and LAG3. Increased co-expression of multiple TCIRs aggravates T cell depletion. Blocking interferons can reverse resistance caused by T cell depletion [116]. Konen and others have found that NTRK is upregulated by anti-PD-1 therapy. NTRK abnormally activates the JAK-STAT signaling pathway, upregulates the expression of multiple inhibitory receptors on T cell surfaces, including PD-1, and promotes T cell depletion [117]. Tregs also promote the expression of CD8^+^ T cell depletion-related gene expression via IL-10 and IL-35. Sawant et al. found that IL-10 regulates the STAT pathway and IL-35 regulates the STAT1\4 pathway, further altering the expression of BLIMP1 and its target genes. BLIMP1 enhances the expression of inhibitory receptors in T cells and promotes T cell depletion (Fig. 5) [118].

#### Resistance caused by changes in PD-L1 expression

The response to PD-1/PD-L1 blockade therapy is better in tumors with PD-L1-positive expression [9]. Both membrane expression and secretion exosomes containing PD-L1 may contribute to resistance. PD-1 blockade therapy can result in the upregulation of PD-L1 expression, causing drug resistance. The insufficient antibodies do not completely block PD-1/PD-L1. Conversely, low PD-L1 expression reduces therapeutic efficacy; this may be explained by other immune escape mechanisms.

The JAK/STAT pathway is critical for PD-L1 expression and drug resistance [119–121]. Because JAK/STAT up-regulates the expression of PD-L1, it also plays an important role in tumor antigen expression. JAK1 is essential for both IFN-γ-mediated immune responses and MHCI/II expression, whereas JAK2 contributes to IFN-γ-induced STAT5 phosphorylation and PD-L1 expression, and mutations disrupt antigen presentation [122]. In addition to the JAK/STAT pathway, other factors cause changes in PD-L1 expression. In large B lymphoma, miR155 binds to the 3′-UTR of PD-L1 to increase its expression and inhibits CD8^+^ T cell activity through the ERK and AKT pathways. Similar effects have been found with miR-142-5p in pancreatic cancer; however, miR-142-5p overexpression inhibits tumor cell PD-L1 expression and enhances tumor immunity [123].

In melanoma, resistance due to JAK1/JAK2 inactivation mutations, leading to recurrence, has been found in a small number of patients [87, 119]. Patients with JAK1/2 mutations can develop drug resistance, irrespective of TMB [124–126]. JAK1/2 regulates the chemokines CXCL9, CXCL10, and CXCL11 [127]. Deletion of the tumor suppressor CDKN2A, one of the most frequently lost tumor suppressor genes in human cancers, increases the likelihood of JAK2 deletion and resistance to immunotherapy [128].

Many factors lead to the adaptive up-regulation of PD-1 and drug resistance. In a mouse model of KP mutant lung cancer, neurotrophic tyrosine receptor kinase 1 (NTRK1) expression increased significantly after treatment with a PD-1 inhibitor, and NTRK1 promoted abnormal JAK1 and STAT3 activation. Excessive JAK/STAT pathway activation leads to PD-L1 up-regulation [117]. In NKT cell lymphoma, after PD-1 blockade, the JAK/STAT pathway is activated via IFN-γ secreted by TILs, promoting PD-L1 expression [48]. In most patients with lung cancer and non-T790 M-mediated epidermal growth factor receptor (EGFR) mutations, the downstream JAK/STAT, AKT/mTOR, and mitogen-activated protein kinase (MAPK)1 pathways are not activated, resulting in unexpressed PD-L1 and resistance to PD-1 blockade therapy [129–135]. However, the JAK pathway also promotes inflammation and other functions in the tumor microenvironment [136]. We cannot rule out the effects of the inflammatory response on PD-L1 and therapeutic efficacy. Mutations in the serine/threonine-protein kinase gene, *BRAF,* in tumors also increase PD-L1 expression and induce drug resistance involving tumor stromal cells. *BRAF* mutations also lead to constitutive activation of the MAPK pathway, enhance the oncogenic activity, increase invasiveness and metastasis, and cause resistance [137].

PD-L1 exosomes have been detected in a variety of cancers, including melanoma and head and neck cancer [119, 138]. High IFN-γ levels are associated with drug resistance [119]. Other studies have shown that the increase in PD-L1 is mainly due to exosomes, rather than membrane expression. Exosomes may even induce the expression of T cell depletion markers. Immunotherapy results in TNF-α production and T cell accumulation in tumors, promotes histone methylase EZH2 activity in melanoma, decreases immunogenicity, silences antigen-presentation, and up-regulates PD-L1 expression. After the inactivation of EZH2, resistance is reversed by the continuous aggregation of CD8^+^ T cells with low PD-1 and IFN-γ levels [139]. In lung adenocarcinoma, EZH2-positive patients show high PD-L1 expression [140]. In mice, TNF can promote EZH2 expression in tumor cells and trigger tumor recurrence [92, 141]. In patients with metastatic melanoma treated with PD-1, *TNF* expression is increased, and there is a strong positive correlation between *TNF* and *PDCD1LG1* (encoding PD-L1). TNF-α increases PD-L1 stability by activating COP9 signal 5 [142].

**Fig. 6:**
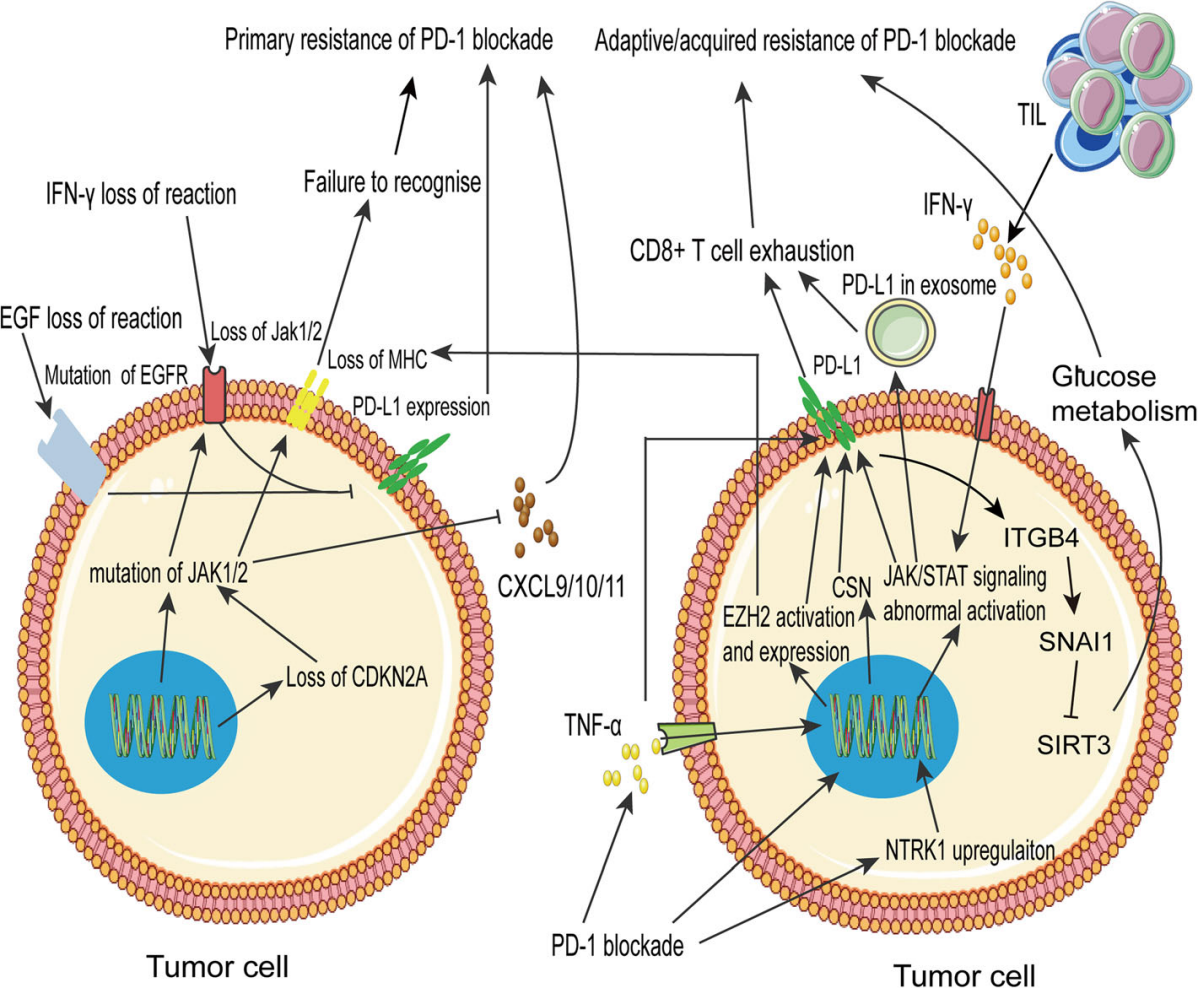
PD-L1 expression changes contribute to resistance. Mutations in the *JAK* and *EGFR* genes result in the loss of PD-L1 expression, affecting antigen presentation. The lack of CXCL9, 10, and 11 expression prevents T cell chemotaxis in the tumor microenvironment, causing primary resistance. After PD-1 blockade, adaptive changes in various genes alter tumor cell metabolism, reduce MHC expression, and upregulate PD-L1, resulting in the inhibition of T cell activity and adaptive resistance

PD-L1 also has a direct effect on tumors. It binds to the surfaces of tumor cells via integrin-binding β4 (ITGB4) and activates the protein kinase/GSK3β signaling pathway, thereby inducing the transcriptional repression of *SNAI1*. SNAI1 regulates *SIRT3*, epithelial-mesenchymal transition-related genes, and glucose metabolism and promotes lymphatic metastasis. That is, PD-L1 promotes tumor growth and metastasis via ITGB4/SNAI1/SIRT3 signaling, and this is one of the main causes of PD-L1 resistance [143]. This suggests that targeting PD-1/PD-L1 in combination with downstream factors, including ITGB4, can enhance the immunological efficacy of PD-1/PD-L1 (Fig. 6).

### Combination therapy to improve the efficacy of PD-1/PD-L1 blockade

Based on the aforementioned mechanisms underlying resistance to PD-1 blockade therapy, we explore candidate targets for combined PD-1 immunotherapy, providing new hope for improving the therapeutic efficacy through increasing T cell proliferation and enhancing immune cell function.

#### Combination therapeutic strategies to enhance T cell activation

Two strategies can enhance T cell activation: enhancing tumor immunogenicity and enhancing the activation of co-stimulatory signals on primitive and memory T cells.

The induction of immunogenic cell death (ICD) has been proposed as an effective way to enhance tumor immunogenicity. Dying tumor cells can express or release extensive immunostimulation damage-associated molecular patterns. This process also releases high mobility box 1 (HMGB1) and ATP to attract and activate APCs. Calreticulin on the surface of dead cells transmits an ‘eat-me’ signal to phagocytic cells to activate macrophages, ultimately leading to enhanced tumor immunogenicity and immune responses. There is a significant synergistic effect between the induction of ICD and PD-1 blockade [144–148]. In addition to ICD, Kim et al. suggested that the restoration of the function of the tumor suppressor p53 can also enhance tumor cell immunogenicity, thereby enhancing the innate and adaptive immune response and counteracting tumor-induced immunosuppression. Additionally, heterogeneous hypersensitivity reactions associated with PD-1 antibodies are alleviated, which can alleviate the side effects of PD-1 treatment [149–153].

Various molecules that enhance co-stimulatory signaling for T cell activation have been identified. Chimeric antigen receptor T cells edited by the CRISPR/Cas9 gene directed against the B2M mutation proposed above can significantly increase anti-tumor activity [154].

**Fig. 7:**
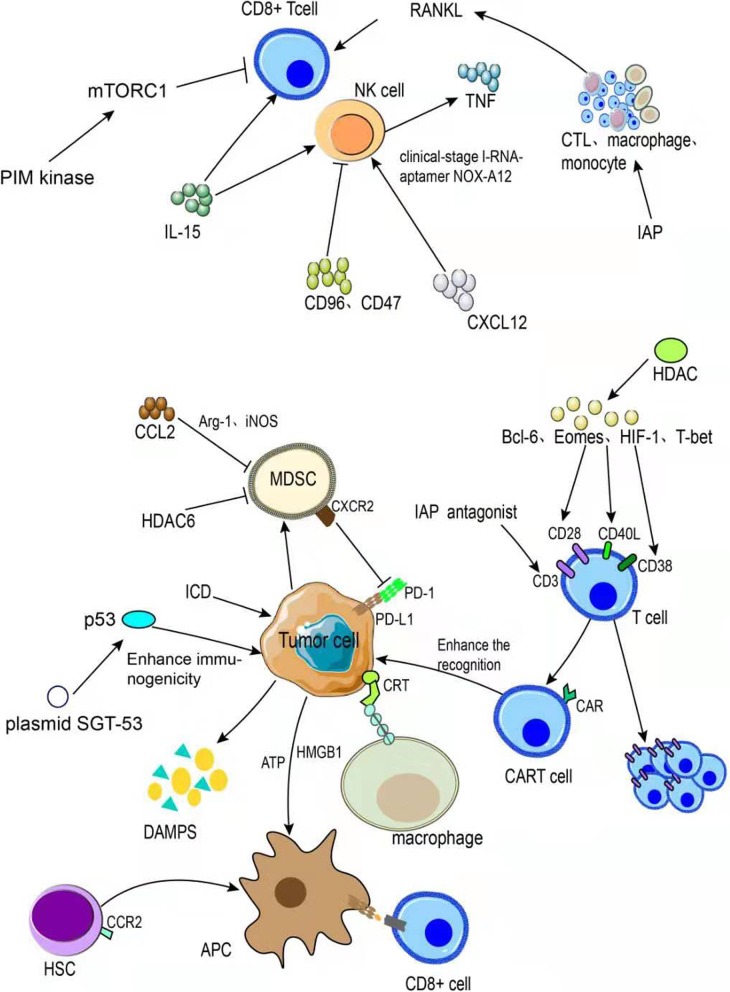
Combination therapeutic strategy to enhance T cell activation and T immune cell function and infiltration. There are approximately five types of combined treatment strategies. Combinations with anti-PD-1/PD-L1 agents can induce better therapeutic effects by inducing immunogenic cell death and restoring the function of the tumor suppressor p53. We summarize the combinations with B2M, HSCs (CCR2+), HDAC, and other cells and molecules. We describe a number of ways to inhibit MDSCs and thereby enhance therapeutic efficacy. Various molecules, including IL-15, CD96, CD47, and CD137 have potential inhibitory effects. We also summarize receptor-mediated and combination therapeutic strategies for the activation of inflammatory pathways and immune cells

Inhibitor of apoptosis protein (IAP) has extensive biological functions, including the regulation of migration, apoptosis, and signal transduction and the promotion of inflammation. IAP antagonists, including Smac mimetics, can enhance the activation and proliferation of effector T cells by enhancing CD3/CD28 co-stimulation [107]. Additionally, bone marrow-derived hematopoietic stem cells expressing type 2 C-C chemokine receptor (CCR2^+^ HSCs) preferentially migrate to tumor tissues and differentiate into APCs in the tumor microenvironment. The presentation of tumor-derived antigens to CD8^+^ T cells overcomes resistance to PD-1 checkpoint blockade [155]. Histone deacetylases (HDAC) are a therapeutic target for a variety of cancers. The inhibition of HDAC6 activates the AKT/mTOR/p65 pathway and up-regulates BCL-6, Eomes, HIF-1, and T-bet, thereby increasing the expression of co-stimulatory molecules (CD28, 41bb, CD40L, OX40, and CD38) and activation of antigen-specific memory T cells [156]. B-type TILs are good prognostic markers for most cancers [157]. Soldevilla et al. proposed that the injection of activated B lymphocytes in combination with anti-PD-1 agents could improve therapeutic efficacy. Combined with anti-PD-1 treatment, it is possible to provide multiple costimulatory ligands in the tumor and activate the systemic anti-tumor immune response, with superior anti-tumor effects (Fig. 7) [158].

#### Combination therapeutic strategy to enhance T immune cell function and infiltration

Activated T cells need to infiltrate the tumor tissue to exert anti-tumor effects, alone or in combination with other immune cells. We next discuss factors that increase the density of T cells in tumor tissues and enhance immune cell function.

The inflammatory response increases following IAP blockade, thereby stimulating CTLs and mononuclear/macrophage TNF production and enhancing tumor cell killing [107]. Blocking IAP acts synergistically with anti-PD-1 treatment to enhance anti-tumor immunity. In addition to IAP, IL-15, CD96, CD47, and CD137 affect immune cell activity and have potential therapeutic applications. When IL-15 is activated, the number and activity of CD8^+^ T and NK cells increase [159]. CD96 regulates the effects of NK cells and metastasis. CD96-deficient CD8^+^ T cells are superior to CD96-sufficient CD8^+^ T cells at suppressing tumors, and the co-expression of CD96 and PD-1 has been detected in both mouse and human TILs, suggesting an immune-inhibitory effect. Blocking CD96 can significantly enhance the interaction between NK and T cells and increase their anti-tumor effect [160]. Blocking CD47 also increases the reactivity of anti-tumor T and NK cells and increases the release of various cytokines, including IFN-γ and IL-6. Moreover, the simultaneous blocking of CD47 and PD-1 can further prevent the immune escape of circulating tumor cell subsets, thereby inhibiting metastasis [161, 162]. Rodríguez-Ruiz et al. proposed that combined anti-PD1 and anti-CD137 treatment increases granzyme-B secreted by CTLs, indicating an improved cytotoxic effect [163]. RANKL, which blocks NF-κB ligands, can increase the anti-metastatic activity of antibodies targeting PD1/PD-L1, and the combination of anti-PD1 and anti-RANKL agents can recruit NK cells to promote the synergy between NK cells and TILs. This increases the secretion of interferon and tumor killing factors [164]. Low PD-L1 expression is also a major cause of poor PD-1 blocking; accordingly, co-inhibitory receptors are a promising area of research. The newly discovered T cell B7 family immune checkpoint, HHLA2, is a co-therapeutic target for PD-L1, improving the number and activity of T cells in the tumor microenvironment [165]. Another co-inhibitory receptor, KLRG1, expressed on late-differentiated effector cells and CD8^+^ T and NK cells, is up-regulated in treated tumor samples, resulting in drug resistance; blocking both KLRG1 and PD-1 can improve outcomes [166]. However, more potent co-inhibitory receptor blockade may not result in a better therapeutic effect. Pai et al. found that combination therapy targeting PD-1 and CTLA-4 induces an excess of IFN-γ and leads to drug resistance. Excess IFN-γ increases IDO and PD-L1 expression. There is a threshold for co-inhibitory receptor blocking, beyond which the effects are reversed [167]. This deserves further exploration, and the dose range for combination therapy should be optimized.

MDSC proliferation is another cause of tumor immune escape. This limits the efficacy of PD-1/PD-L1 blockade. The generation and migration of MDSCs are regulated by multiple chemokines. It is essential to inhibit MDSC proliferation and migration to the tumor microenvironment while blocking PD-1. In children with metastatic sarcoma, the efficacy of PD-1 blockade therapy was significantly improved by treatment with an anti-CXCR2 monoclonal antibody [168]. CCL2 is positively correlated with MDSCs in tumor tissues, suggesting that it promotes MDSC migration to tumor tissues. In tumor-bearing mice, CCL2 expression is significantly increased in the blood and tumor tissues. Anti-CCL2 treatment inhibits the expression of arginase 1 and iNOS, thereby reducing G-MDSC and M-MDSC in and around the tumor. Combination therapy can increase CD4^+^ and CD8^+^ T cell infiltration and prolong the survival of tumor-bearing mice [169]. Furthermore, the inhibition of HDAC6 significantly reduces HLA-DR-Low/CD11b^+^CD33^+^ MDSCs in the tumor microenvironment [156]. The chemokine CXCL12, an immunosuppressive molecule, combined with clinical-stage I-RNA-aptamer NOX-A12, increases the infiltration of T and NK cells in solid tumors [66].

The inhibition of PIM kinase may address the T cell depletion issue. PIM kinases are a family of serine/threonine kinases that promote cell cycle transition, cell growth, mTORC1 activity, and the ability of T cells to inhibit tumors. PIM kinase inhibition upregulates the expression of genes involved in the inhibition of glycolysis and reduces CD38 expression in negatively regulated T cell metabolism. The inhibition of PIM can increase the tolerance and persistence of T cells in the tumor microenvironment, and the combined effect with blocking PD-1 can significantly improve efficacy (Fig. 7) [170].

#### Combination therapeutic strategy for combined chemoradiotherapy

In addition to the above-mentioned proposed strategies to enhance efficacy, we must also discuss chemoradiotherapy combined with anti-PD1 immunotherapy, which has been implemented in clinical practice. Clinical trials have shown that this strategy have achieved satisfactory results in NSCLC, gastric, (triple-negative) breast, recurrent nasopharyngeal, and rectal cancers, hematological malignancies, and other tumors [171–178]. The combined effects of chemoradiotherapy are due to the enhanced immunogenicity of tumor cells, antigen presentation, and recognition of tumor cells by T cells. Chemoradiation increases the tumor mutation load amd exposes antigens [179]. Simultaneously, the tumor microenvironment becomes more conducive to anti-tumor immunity. On the one hand, there are changes in cellular components in the microenvironment, including increased inflammatory cells and decreased MDSCs [180]. On the other hand, radiotherapy can cause changes in gene expression in various cells in the tumor microenvironment. Some studies have found that radiation induces upregulation of MHCI, intercellular adhesion molecule 1 (ICAM-1), NKG2D ligand (NKG2DL), death receptor Fas, and costimulatory molecule CD80 on tumor cells, which enhances both antigen presentation and T cell recognition [181]. Other studies have found that in NSCLC, radiotherapy can adaptively increase the expression of PD-L in tumor cells. This may also be one of the mechanisms [171]. However, two clinical trials have shown that PD-1 immunotherapy after radiation therapy can cause an excessive immune response, as seen in the adverse effects of combination therapy in obesity-related malignancies, including esophageal adenocarcinoma [182, 183]. This may be because the relationship between radiation and the immune system is complex and multifactorial, and is related to the dose and type of radiation and the type of immune cells [181]. Furthermore, this process induces an inflammatory response, and different degrees of inflammatory response may lead to different outcomes. Therefore, it is essential to clarify the basic combination therapy mechanism further, and the specific scheme and dosage of the combination in the clinic need to be determined (Table 2).

**Table 2:** Combinations of immunological checkpoint inhibitors in US clinical trials

Immunological checkpoint inhibitor	Combined drug	Application	Number of volunteers	OS (months)	Rate of OS (at 6 months)	ORR (%)	DOR (months)	PFS (months)
pembrolizumab	Epacadostat [168]	Unresectable or metastatic melanoma	354	–	84.1	34.2	–	4.70
	Pomalidomide+Dexamethasone [169]	Refractory or relapsed and refractory multiple myeloma	126	21.0 (14.2-NA)	–	–	–	5.7
nivolumab	Ipilimumab [170, 171]	Previously untreated advanced melanoma	313	–	0.86	57.6	–	11.50
		Previously untreated advanced or metastatic renal cell carcinoma	550	–	–	38.7	–	12.42
ipilimumab	Sargramostim [172]	stage III or stage IV melanoma untreatable by surgery	123	17.5 (14.9-NA)	–	–	–	3.10
	Dacarbazine [173]	untreated unresectable stage III or IV melanoma	250	11.17	–	–	19.3	2.76
	Paclitaxel/ Carboplatin [174]	Lung cancer—non small cell squamous	388	13.37	–	–	–	5.55
	Nab-Paclitaxel + Carboplatin [175]	Non-squamous non-Small cell lung cancer	483	18.6	–	–	–	7.00
atezolizumab	Carboplatin + Etoposide [176]	Untreated extensive-stage small cell lung cancer	201	12.3	–	–	–	5.2
	Cobimetinib [177]	Metastatic colorectal adenocarcinoma	183	8.87	–	–	1.97	1.91
	Bevacizumab [178]	Renal cell carcinoma	178	–	–	–	–	8.90

#### Outlook

Despite the unique advantages of tumor immunotherapy demonstrated by recent research, this approach is still highly limited in clinical settings due to drug resistance and high costs. We review common bio-predictive markers and therapies and discuss the molecular mechanisms underlying resistance to PD1/PD-L1 blockade therapy. Based on these mechanisms, we describe promising drugs and potential molecular targets for combination therapy.

Although the biomarkers that can be used for prediction are described above, they still have significant uncertainties in the clinic. Error in predicting PD-L1 expression is mainly related to tumor heterogeneity and differences among the monoclonal antibodies used for detection [23, 184]. At present, IHC is primarily used to measure PD-1 expression; however, other antibodies, including E1L3N, SP142, and SP263, are also used [185]. There is no standard method for quantification, which is a problem that needs to be solved. Morales-Betanzos et al. established a targeted mass spectrometry platform that can quantify the expression of PD-L1. Regarding tumor heterogeneity, the minimum tumor area that can determine the PD-L1 prediction evaluation must be elucidated [186, 187]. The International TILs Working Group provides a standardized method for pre-treatment tumor TIL testing, comprising a visual assessment of H&E stained sections [188]. Although it has limitations in macrophage detection, it has been widely used in many clinical applications. Furthermore, more practical predictive indicators, such as microbial taxa in the intestines, should be identified in addition to the development of accurate detection methods.

Furthermore, the development of research and detection methods for molecular markers in the blood is of considerable significance because the extraction of peripheral blood for detection has the advantages of being simple and easy to perform and less invasive to the patient. This is an advantage that traditional pathological examinations do not have and should be focused on.

In general, the precise mechanisms underlying drug resistance to PD-1 treatment and appropriate therapeutic strategies are still unclear. Many studies have suggested that high PD-1/PD-L1 expression predicts a good prognosis, but tumors can also develop drug resistance by adaptively up-regulating PD-L1 expression during therapy. The level of PD-L1 is not proportional to the therapeutic effect, and optimal treatment strategies are still needed [167]. We believe that the detection of PD-L1 expression is critical for PD-1 blockade therapy. First, the expression of PD-L1 should be detected to identify whether the tumor is suitable for PD-1 blockade therapy. During treatment, dynamic changes in PD-L1 expression should be detected. Additionally, resistance to PD-1 blockade is caused by exosome PD-L1 secretion. This resistance is caused not only by promoting the expression of PD-L1, but also by the direct binding of PD-L1 exosomes to anti-PD-L1 antibodies. Tumor- and immune-cell-derived PD-L1 exosomes can inhibit tumor progression by promoting antigen presentation and regulating immune function. However, studies are currently focusing on its impact on tumor progression; therefore, the study of exosomes must be more comprehensive [2]. To detect changes in PD-L1 expression and guide precision medicine, more accurate detection methods are needed [189]. More generally, the membrane and exosome expression of PD-L1 should be dynamically monitored. In addition to the effects of PD-L1 expression on drug resistance mechanisms, it has recently been discovered that certain molecular targets already used in cancer treatment also affect the efficacy of immunotherapy, leading to the development of resistance to PD-1/PD-L1 blockade therapy. In addition to TNF-a and IFN-γ mentioned above, there are many inflammatory factors, including IL-6, IL-17, and EGF, that play an important role in the PD-1/PD-L1 signaling pathway, which is in line with the idea that inflammation promotes tumorigenesis as opposed to metastasis. These inflammatory factors have potential effects on tumor immune escape, providing new targets for combined immunotherapy [182]. As a research hotspot in immunotherapy, neoantigen vaccines have been used to screen and identify highly exogenous neoantigens by sequencing the entire exons of tumor cells to activate immune responses. These neoantigens have also been combined with PD-1/PD-L1 blockade therapy with good effects [172].

The combination of PD-1 and other immune checkpoint blockade is a potentially effective treatment strategy. Increased blockade does not predict a better effect; there is a threshold, after which the opposite effects are observed [189]. In short, the human immune system represents a precise balance among various molecules, immune cells, and effectors. The role of any single pathway cannot be considered in isolation.

In addition to immune checkpoints and immune system activity, synergistic treatment approaches, including strategies to activate tumor cell autophagy, inhibit tumor angiogenesis, and inhibit mesenchymal transition, can also improve the efficacy of PD-1/PD-L1 blockade therapy. We should broaden our thinking to the perspective of the tumor itself, e.g., inhibiting nutrient supply, growth, and metastasis, and consider combined approaches with immunotherapy to achieve better results.

## Conclusions

Despite the success of PD-1/PD-L1 treatment, its practical application is still limited. To determine whether a patient may benefit from anti-PD-1 treatment and reduce the burden on patients, PD-1/PD-L1 expression and predictive indicators should be dynamically monitored throughout the treatment process. Established prediction molecules are still insufficient, and improved prediction methods are needed. To address drug resistance, a more systematic research approach should be adopted, beyond studies of particular target molecules. The limits of various drugs and the potential for excessive doses should be considered. Finally, we should actively search for joint treatment strategies to expand the scope and effectiveness of immunotherapy.

## Data Availability

Not application.

## Abbreviations

ANC: Absolute Neutrophil Counts
APC: Antigen-Presenting Cell
ATRA: All-Trans-Retinoic Acid
CCR2+ HSCs: Hematopoietic Stem Cells Expressing Type 2 C-C Chemokine Receptor
CRC: Colorectal Cancer
EGFR: Epidermal Growth Factor Receptor
G-CSF: Granulocyte Colony-Stimulating Factor
HDAC: Histone Deacetylase
ICD: Immunogenic Cell Death
MDSC: Myeloid-Derived Suppressor Cell
MHC: Major Histocompatibility Complex
MMR: Mismatch Repair
MMR-D: Mismatch Repair Defects
MSI-H: High Microsatellite Instability
NLR: Neutrophil-to-Lymphocyte Ratio
NSCLC: Non-Small-Cell Lung Cancer
OS: Overall Survival
RCC: Renal Cell Cancer
TCIR: T Cell Inhibitory Receptors
TIL: Tumor-Infiltrating Lymphocytes
TMB: Tumor Mutation Burden
