# Correction to: Predictive biomarkers and mechanisms underlying resistance to PD1/PD-L1 blockade cancer immunotherapy

**DOI:** 10.1186/s12943-020-01148-y

**Published:** 2020-02-14

**Authors:** Daixi Ren, Yuze Hua, Boyao Yu, Xin Ye, Ziheng He, Chunwei Li, Jie Wang, Yongzhen Mo, Xiaoxu Wei, Yunhua Chen, Yujuan Zhou, Qianjin Liao, Hui Wang, Bo Xiang, Ming Zhou, Xiaoling Li, Guiyuan Li, Yong Li, Zhaoyang Zeng, Wei Xiong

**Affiliations:** 1 NHC Key Laboratory of Carcinogenesis and Hunan Key Laboratory of Translational Radiation Oncology, Hunan Cancer Hospital and The Affiliated Cancer Hospital, Xiangya School of Medicine, Central South University, Changsha, Hunan China; 2 Key Laboratory of Carcinogenesis and Cancer Invasion of the Chinese Ministry of Education, Cancer Research Institute and School of Basic Medical Science, Central South University, Changsha, Hunan China; 3 Hunan Key Laboratory of Nonresolving Inflammation and Cancer, Disease Genome Research Center, The Third Xiangya Hospital, Central South University, Changsha, Hunan China; 4 Department of Medicine, Dan L Duncan Comprehensive Cancer Center, Baylor College of Medicine, Houston, TX USA

**Correction to: Molecular Cancer (2020) 19: 19**

**https://doi.org/10.1186/s12943-020-1144-6**

After publication of the article [1], authors found out that Fig. 1 was inadvertently replaced. Correct Fig. 1 version is provided on this paper.

**Fig. 1:**
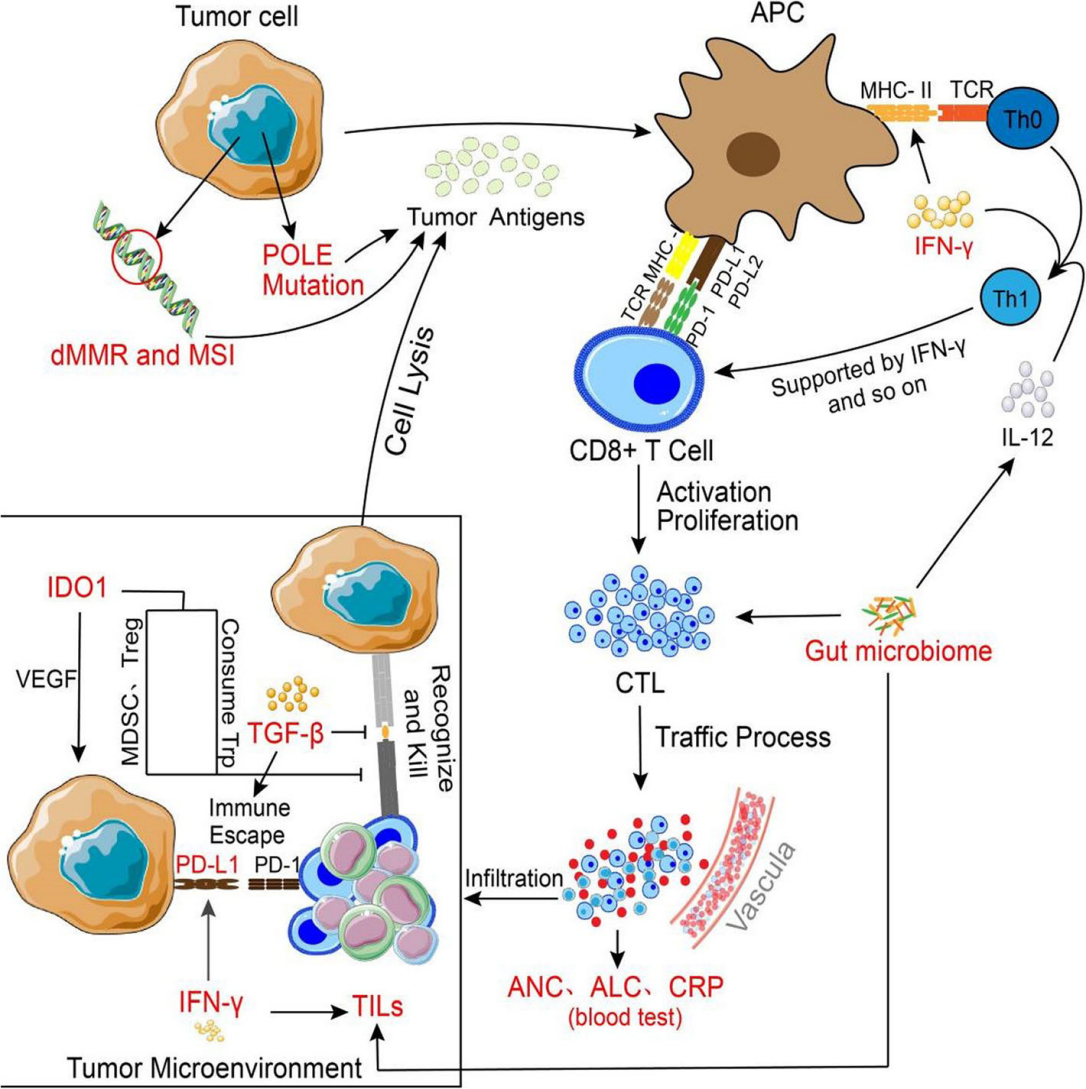
Summary of biomarkers of the response to anti-PD-1/PD-L1 immunotherapy
